# Intimate Partner Violence and Abuse: A Qualitative Exploration of UK Military Personnel and Civilian Partner Experiences

**DOI:** 10.1007/s10896-022-00446-x

**Published:** 2022-11-04

**Authors:** Rebecca Lane, Rachael Gribble, Filipa Alves-Costa, Anna Taylor, Louise M Howard, Nicola T Fear, Deirdre MacManus

**Affiliations:** 1 Department of Forensic and Neurodevelopmental Sciences, Institute of Psychiatry, Psychology and Neuroscience, King’s College London, 16 De Crespigny Park, SE5 8AB Camberwell, London, UK; 2 King’s Centre for Military Health Research, Weston Education Centre, King’s College London, 10 Cutcombe Road, SE5 9RJ London, UK; 3 Barnet, Enfield & Haringey Mental Health NHS Trust (North London Forensic Service), London, UK; 4 Research Department of Clinical, Educational and Health Psychology, University College London, 1-19 Torrington Place, WC1E 7HB London, UK; 5 Section of Women’s Mental Health, Institute of Psychiatry, Psychology and Neuroscience, King’s College London, Dr Crespigny Park, SE5 8AF London, UK

**Keywords:** Intimate Partner Violence and Abuse (IPVA), UK Military personnel, Civilian spouses, perpetration, victimisation

## Abstract

**Purpose:**

The prevalence of Intimate Partner Violence and Abuse (IPVA) perpetration and victimisation has been found to be higher in serving and ex-serving military samples compared to civilians. Despite this, there is a lack of qualitative research exploring the IPVA experiences of couples in which one or both partners are serving or have served in the military. This qualitative study aimed to explore IPVA experiences within the UK military community from the perspective of serving and ex-serving military personnel and civilian partners of UK military personnel.

**Method:**

One-to-one telephone interviews were conducted with 40 serving and ex-serving military personnel (29 male, 11 female) and 25 female civilian partners. Data was analysed using thematic analysis.

**Results:**

Four superordinate themes were derived: (1) patterns and directions of IPVA, (2) types of IPVA, (3) perceived drivers of IPVA and (4) perceived impact of IPVA. The findings point to frequent bidirectional abuse in part driven by poor communication and emotion regulation, whilst also highlighting the experiences of severe IPVA victimisation of civilian partners by military personnel motivated by power and control. Perceived drivers of both IPVA perpetration and victimisation include military factors borne of military culture or training, alcohol and mental health difficulties.

**Conclusion:**

These results highlight the role of cultural norms, as well as the role of emotion dysregulation, poor communication skills and mental health difficulties in explaining and perpetuating abuse within ecological theoretical frameworks of violence among couples within which one or both partners are serving or ex-serving military personnel.

Intimate Partner Violence and Abuse (IPVA) includes emotional abuse (e.g. belittling, humiliating), psychological abuse (e.g. threatening behaviour, verbal aggression) and physical or sexual violence between current and former partners (WHO, 2012). The impact of IPVA can be severe and widespread, marking it is as a public health issue and global health priority (WHO, 2012). In addition to affecting the physical and mental health of victim-survivors (Campbell, 2002), IPVA can affect social and occupational functioning (Hines & Douglas, 2018; Johnson et al., 2014) and have a long-term negative impact on children who are witnesses to domestic abuse (Domoney & Trevillion, 2021; Jouriles & McDonald, 2015). There is a cost to society of IPVA, with an estimated £66 billion spent on victim-survivors of domestic abuse in England and Wales between March 2016 and March 2017 (Oliver et al., 2019).

Our understanding of IPVA typologies, patterns and drivers has advanced greatly in the past 25 years (Gibbs et al., 2020). The ecological framework for violence (WHO, 2002), which includes partner violence, views interpersonal violence as the outcome of the interaction among many factors across four levels—the individual, the relationship, the community, and the societal. In addition to general risk factors, such as socioeconomic status, childhood trauma, gender inequality, Gibbs and colleagues (2020) view involvement in armed conflict as a significant additional driver of IPVA in some communities due in part to increased risk factors at an individual level through exposure to traumatic events and the chronic stress of living under constant threat of attack resulting in worsened mental health and substance misuse (Gibbs et al., 2020). In addition, couples in which one or both partners are serving in the military can be exposed to unique stressors, including frequent geographical relocations and periods of separation, which may negatively impact relationships and increase risk of IPVA (Clark & Messer, 2006; McLeland et al., 2008; Rentz et al., 2006). Evidence suggests that stressed couples are more likely to be aggressive couples (Eckhardt & Parrot, 2017). Chronic external stresses interact with individuals’ dispositional and regulatory deficiencies, and can result in a spill-over of these stresses into the relationship. Drawing on theories of social learning (Bandura, 1978), it has also been suggested that socialisation into military culture and attitudes, which include a defined hierarchy and the legitimisation of aggression within military contexts, can result in “occupational violence spill-over” into the family home (Bradley, 2007; Trevillion et al., 2015). There is evidence to suggest that both IPVA perpetration and victimisation is more prevalent among serving and ex-serving military personnel compared to the general population (Kwan et al., 2020; Rentz et al., 2006; Sparrow et al., 2020), even after adjusting for sociodemographic characteristics (MacManus et al., 2022). In-depth exploration of the types, patterns, impact of, and motivations for, IPVA behaviours perpetrated or experienced by serving and ex-serving military personnel or their partners is lacking, with no such research on UK military communities published to date. Although quantitative data (e.g. MacManus et al., 2022) allows for meaningful comparison of prevalence of IPVA within a military population with the general population and is instrumental in establishing potential risk factors, it is more difficult to explore the nuanced ways in which such factors might affect experiences of IPVA, what triggers may exist and how these may differ between military personnel and their civilian partners.

There remains a need to better understand the lived experiences of serving and ex-serving military personnel and their partners to understand how to best protect victim-survivors of IPVA and tailor interventions for those who engage in abusive behaviours. This is also essential to inform the responses by the Armed Forces, the UK Government, and relevant charities and state services. The current study aimed to qualitatively explore the IPVA experiences of serving and ex-serving UK military personnel and civilian partners to address these gaps. In particular, we wanted to understand the patterns, types and severity of abusive behaviours experienced within relationships and how these experiences affected the participants and their family. To allow us to describe and compare the range of experiences of IPVA perpetration and victimisation within couples in which one or both partners are serving or ex-serving military personnel, we utilised data from interviews with serving and ex-serving military personnel and civilian partners of serving or ex-serving military personnel who reported IPVA behaviours in their relationship(s).

## Methods

### Study Design

The research forms part of a wider mixed-methods research programme aiming to better understand the experiences and complexities of IPVA within the UK Armed Forces community and allowing for triangulation of findings (Alves-Costa et al., 2021, 2022; Lane et al., 2022, under under review-a, under review-b; MacManus et al., 2022). Using a critical realist approach, this paper draws on qualitative data from two samples exploring the experiences and impact of IPVA in relationships in which one or both partners are serving or ex-serving military personnel. Samples were recruited in parallel and had comparable interview schedules (Study 1 and Study 2) and were combined for the purpose of this study in order to provide as comprehensive an account of IPVA in military communities as possible. The first sample (Study 1) included serving and ex-serving military personnel who had reported either perpetrating or experiencing abusive behaviours in the past 12 months and the second sample (Study 2) included civilian partners who had been in an abusive relationship with a serving or ex-serving member of the UK Armed Forces.

### Definitions

IPVA is defined as “*any behaviour within an intimate relationship that causes physical, psychological or sexual harm to those in the relationship*” (WHO, 2012, p1). In this paper, the term ‘bidirectional abuse’ is used to describe cases where participants report relationships with mutual violence, whereby both partners engaged in abusive behaviour toward the other. This may be symmetrical or asymmetrical in terms of severity, but both partners are considered to instigate abusive behaviours and the behaviours are not suggestive of perpetration with retaliation or resistance. We use the term ‘unidirectional’ abuse in cases where participants report perpetrating IPVA towards their partner or being a victim-survivor with no reports of bidirectional violence. Unidirectional abuse may occur with or without instances of retaliation or resistance.

Given that this study focuses on relationships and relationship behaviours occurring during and after military service, the terms ’military personnel’ and personnel will be used to refer to the experiences and perceptions of both serving (active duty) and ex-serving (veteran) personnel in presenting the findings. Differences between serving and ex-serving personnel are pulled out where relevant, although please note serving status is captured at time of interview therefore an ex-serving military respondent may speak of relationships which occurred whilst serving. In addition, recent quantitative research highlights no significant difference in reporting of IPVA perpetration or victimisation between serving and ex-serving UK military personnel, and many risk factors for IPVA perpetration and victimisation overlap (MacManus et al., 2022) and are likely relevant both during and after leaving Service, such socialisation into military culture or trauma exposure on deployment (Lane et al., under review-b; MacManus et al., 2022). Participants who identify as civilian victim-survivors will be referred to throughout the findings as civilian respondents. Their serving or ex-serving military (ex)partners will be referred to as ‘military partners’. The term participants will be used to describe the whole sample where relevant.

### Recruitment

Military personnel were recruited from a sub-sample of respondents to Phase 3 of the King’s Centre for Military Health (KCMHR) Health and Well-being Cohort Study (Stevelink et al., 2018). Within the self-completed survey, serving and ex-serving personnel reported on experiences of victimisation and perpetration of IPVA in the past year, including psychological, emotional, physical and sexual abuse (MacManus et al., 2022). 266 serving or ex-serving military personnel (188 men and 78 women) who reported IPVA perpetration and/or victimisation, and consented to be followed up, were invited to take part.

Individuals who identified as civilian victim-survivors of IPVA occurring during relationships with military or ex-military personnel were also eligible for inclusion. Civilian respondents were recruited via advertisements through national military and civilian welfare support charities, clinical services for serving military personnel and their families (including military base GPs and welfare services) or services for veterans and their families, and specific support organisations for victim-survivors of IPVA. Although civilian respondents were recruited based on their experiences of victimisation in a relationship with a military partner, all participants were asked about victimisation and perpetration of abuse or retaliation within relationships at interview.

Recruitment for both groups was open to all genders, ethnicities and sexual orientations. Prior to involvement, participants received study information and provided written consent. Semi-structured one-to-one telephone interviews lasting between one to two hours were conducted between January to August 2018. All interviews were audiotaped/recorded and transcribed verbatim before anonymisation. Participants were given £25 as compensation for their time.

### Analysis

Interviews were analysed using Reflexive Thematic Analysis (Braun & Clarke, 2020), a method well-suited to research questions regarding lived experiences. It allows for exploration of patterns of meaning across the data using both deductive themes informed by theory and literature, and inductive, data-driven themes. This analysis approach allows for immersion in the data through the inductive coding process, whilst acknowledging the lens provided by existing frameworks and theory. In this study, WHO definitions of IPVA, ecological frameworks of violence, and occupation violence spill-over theories were used as scaffolding for data-driven themes. Following the transcription and *familiarisation* with the data, an initial *coding framework* was constructed through a process of open coding (Braun & Clarke, 2020). This initial framework was applied to the remaining transcripts, *generating initial themes and subthemes*. Coding was conducted by two authors (RL, RG). The first coder (RG) coding 50% of the data, which was reviewed by the second coder (RL), who then adapted and added to the early theme development using the remaining data. The coding frame was *reviewed* and refined through progressive iterations and discussions within the research team, and themes and subthemes were *defined*. All themes were data-driven, and range from descriptive to more latent themes suited to answer the research questions posed. Given the descriptive and potentially identifying nature of the themes and subthemes, we have opted not to present quotes to protect participant confidentiality in some areas. Comparisons across sub-groups (e.g., gender, reported IPVA experiences, branch of Service, serving status, rank, military/civilian) were considered and reported when observed. Data analysis and management was supported by QSR Nvivo software (QSR International Pty Ltd., 2020).

### Patient and Public Involvement (PPI)

The research programme benefitted from consultation and feedback from experts by experience and by training. A PPI group was developed involving consultation with professionals (military researchers, IPVA researchers and services, mental health researchers and services, members of the UK Armed Forces) and civilians with personal experience of abuse by serving or ex-serving military partners. Feedback from this group helped inform the interview protocols and refine the frameworks, decreasing the possibility for researcher bias. PPI events were also organised to gain feedback on the findings, which allowed the results to be verified and validated.

### Reflexivity Statement

It is important to reflect that all authors of this paper are White European, female, have never served in any Armed Forces, and have undertaken postgraduate study. Authors have no current or previous affiliations to the UK Ministry of Defence or military. It is possible that author characteristics and pre-conceptions of the military and/or of IPVA have affected the way the interviews were conducted and the analysis was approached, and influenced participant responses. However, the non-military serving status of interviewers was also considered likely to reduce barriers to disclosing issues with the military institution.

### Ethics

Ethical Committee approval for interviews with serving/ex-serving personnel was obtained from the UK Ministry of Defence Research Ethics Committee (823/MODREC/17). Ethical Committee approval for interviews with civilian partners was granted by the King’s College London Research Ethics Subcommittee (REF HR-17/18-5356). Due to the sensitive nature of the interviews, a risk management plan was developed. All participants were offered the opportunity to discuss any concerns following their interview with an independent clinician and were signposted to support services.

### Participants

The sample (*n* = 65) included serving and ex-serving military personnel (*n* = 40) and civilians in current or prior relationships with serving or ex-serving military personnel (*n* = 25). Demographics and military characteristics of military personnel are presented in Table 1. The majority of military respondents described themselves as White or White British. Most personnel were male; between 35 and 50 years of age; reported relationships with civilian partners; were in the Army; were ex-serving at time of interview; were/or had been Regular military personnel; were Non-Commissioned Officers (NCOs); and had previously deployed. Two participants described their experiences within same-sex relationships. Most military personnel spoke of multiple relationships with a range of patterns, severity and types of IPVA across their lifetime.

**Table 1:** Military personnel demographics, relationship information and military characteristics

	*n*
**Gender**	
*Male*	29
*Female*	11
**Age** (years)	
*< 35*	7
*35–49*	22
*50+*	11
**Ethnicity**	
*Minority ethnic group*	4
*White*	36
**IPVA reported** *	
*Reporting IPVA victimisation*	27
*Reporting IPVA perpetration*	17
*Reporting bidirectional IPVA*	29
**Dual military relationship reported**	
*Yes*	16
*No*	24
**Branch**	
*Royal Navy/Royal Marines*	7
*Royal Air Force (RAF)*	11
*Army*	22
**Serving status**	
*Ex-serving (veteran)*	31
*Serving*	9
**Engagement status**	
*Regular*	33
*Reservist*	7
**Rank** (at time of interview or leaving Service)	
*Officer*	7
*Non-Commissioned Officer (NCO)*	30
*Other rank*	3
**Length of service** (years)	
*5 to 14*	19
*15 to 24*	11
*25+*	10
**Deployment experience** **	
*Deployed*	35
*Not deployed*	5

*Some military respondents reported different IPVA patterns across different relationships. As such, these are not mutually exclusive.

**Deployment experience does not include detail on whether military personnel held combat roles on deployment, although military respondent narratives would suggest this was common.

Demographics of civilian respondents and military characteristics of their military partners are presented in Table 2. All civilian respondents in the study were women in heterosexual relationships. The majority of civilian respondents described themselves as White British and were between 35 and 49 years of age. At the time of interview, all but one civilian respondent were no longer in an abusive relationship with a currently serving or ex-serving member of the Armed Forces. Most military partners of civilian respondents were in the Army, were ex-serving, and were NCOs. All had previously deployed and served as Regular personnel, though some served with the Reserves before or after their Regular service. Some participants reported the military characteristics of partners at the time of interview; for others, this reflected their partners’ military characteristics during the relationship or at the point of leaving Service. In addition, some military partners served across branches. Differences according to personnel military characteristics such as Service and rank were explored within the data, however due to these complexities meaningful comparisons were restricted.

**Table 2:** Civilian respondent demographics and military characteristics of (ex)partners

	*n*
**Age** (years)	
*< 35*	6
*35–49*	12
*50+*	7
**Ethnicity**	
*Minority ethnic group*	3
*White*	*22*
**Branch** *	
*Royal Navy/Royal Marines*	6
*Royal Air Force*	2
*Army*	21
**Serving status**	
*Ex-serving (veteran)*	16
*Serving*	11
**Engagement status** *	
*Regular*	27
*Reservist*	4
**Rank**	
*Officer*	3
*Non-Commissioned Officer (NCO)*	14
*Other rank*	8
*Unknown*	2
**Length of service (**years)	
*5 to 14*	11
*15 to 24*	11
*25+*	2
*Not known*	3
Deployment experience**	
*Deployed*	27
*Not deployed*	0

Note: Two civilian respondents reported more than one abusive relationship with a serving or ex-serving military partner, with the total sample reporting on 27 abusive relationships with military personnel.

*These groups aren’t mutually exclusive. Some military partners were reported to serve in multiple Service branches and as both regular and reservist military personnel.

**Deployment experience does not include detail on whether military personnel held combat roles on deployment, although civilian respondent narratives would suggest this was common.

While many military personnel were ex-serving at time of interview and many military partners of civilian respondents were reported to be ex-serving, participants spoke of relationships which spanned the military person’s career in the UK Armed Forces (i.e. before, during and after military service).

## Findings

Four primary themes were derived from the data which describe experiences of IPVA among serving and ex-serving military personnel and civilian respondents (see Table 3). Theme 1 describes the *patterns of abuse* experienced by participants, exploring the direction and severity of abuse within their relationships, how they resolved conflict and how the abuse progressed over time. Theme 2 describes the *types of abuse reported*, covering participant accounts of emotional and psychological abuse, controlling behaviours, physical abuse and sexual abuse. Theme 3 presents the *perceived drivers of abuse*, including motivations and triggers identified in participant narratives. Theme 4 explores participants’ perceptions of *the impact of abuse* on their or their partner’s physical or mental health, their parenting and on their children, and on their occupational and social functioning.

**Table 3:** Themes and subthemes derived from thematic analysis

Themes	Subthemes
1. *Patterns of abuse*	i. *Direction of abuse and mutuality* ii. *Severity* iii. *Progression over time*
2. *Types of abuse reported*	i. *Emotional and Psychological IPVA* ii. *Controlling behaviours* iii. *Physical IPVA* iv. *Sexual IPVA*
3. *Perceived drivers of abuse*	i. *Motivations* ii. *Triggers* iii. *Conflict resolution*
4. *Perceived impact of abuse*	i. *Impact on physical health* ii. *Impact on mental health* iii. *Impact on parenting and children* iv. *Impact on occupational and social functioning*

### Theme 1: Patterns of Abuse

**Direction of abuse and mutuality.** Most military personnel reported having experienced relationships in which bidirectional abuse occurred between partners; this was particularly prominent among dual military couples. Most also reported experiences of unilateral IPVA victimisation in one or more relationships and a large minority reported unilateral perpetration of abusive behaviours (see Table 1). All military personnel who reported perpetration of abuse had previously deployed. No gender or other group differences (such as by Service Branch, rank or engagement or serving status) in the experiences of IPVA were noted. Civilian respondents all described being the victim-survivor of unidirectional abuse perpetrated by their military partner. Only a minority reported retaliation, mostly verbal aggression, in response to the abuse experienced, but such behaviour was not considered commensurate with bidirectional abuse.

**Severity.** While civilian respondents all described frequent moderate to severe experiences of unilateral IPVA victimisation perpetrated by their military partners, the majority of military personnel reported less severe IPVA experiences (such as verbal aggression, lower level controlling behaviours and emotional abuse). Some military personnel appeared to minimise the abuse within their relationships or were more reluctant to disclose details in interviews compared with civilian respondents. However, some military personnel did report experiencing or perpetrating frequent or severe abuse. Reports of more severe experiences of unilateral victimisation or bidirectional abuse were made by both male and female military personnel. Severe reports of IPVA perpetration were more prominent among male personnel.

**Progression over time.** Many military personnel described how the abusive behaviours within their relationships increased over time and were more severe towards the end of their relationship. A minority described the abuse to be present from the early days of their relationship and to remain stable over the course of their relationship. Some reported that behaviours within their relationships improved over time, for instance as they got older, left Service and spent more time together, or learnt better conflict resolution skills.

*[The arguments] definitely escalated over the years that we were together. So what probably started with me just being upset and trying to understand behaviours, then gradually the frustration and the anger came in, and then I would say the violence – [partner’s] outbursts and my outbursts – was the last few years. [P16; Female Army personnel; Dual military relationship; bidirectional abuse reported]*

*Whereas before the argument […] would have carried on, it could be for quite a few days, whereas now we are in a position where, if we do argue, and it is not the severity that we used to before. […] straightaway afterwards, basically, apologising to each other or accepting what the other one said. [P10; Male Army personnel; Dual military couple; bidirectional abuse reported]*

Similarly, most civilian respondents described a pattern of escalating abuse perpetrated by their military partner, with less frequent and less severe forms of psychological and emotional abuse and controlling behaviours in the beginning of the relationship which escalated in frequency, intensity and severity (see Theme 3, Subtheme 2 ‘*Triggers’* for further examples).

In the beginning [(ex)partner exerting control] was only every now and again, and then it would be the odd text message. But, as it got to the last year, year-and-a-half of the relationship, it was incessant; it didn’t stop. (P7; Civilian partner of military personnel)

Most civilian respondents described the relationship to end in a context of heightened abuse, often physical. While for some civilian respondents, the onset of the abuse was easily identified, for others there was a sense that the slow escalation made recognition of the gravity of the abuse difficult. In contrast to military personnel, some civilian respondents also described ongoing abuse post-separation, as depicted in the example below.

[During the divorce process], my ex-husband kept ringing me, kept turning up at my parents’ house. He would ring me or message me and say that he was going to wait outside of where I was working at the time and kill me or kidnap me. There were lots of threats, lots of abusive messages [for] about a year. (P19; Civilian partner of military personnel)

### Theme 2: Types of Abuse

**Emotional and Psychological IPVA.** Emotional abuse (e.g. humiliation, belittling) and psychological abuse (e.g. verbal aggression, threats) were the types of IPVA most commonly shared by military personnel. Both perpetration of emotional abuse and emotional abuse victimisation were reported by male and female personnel. Of note, military personnel described a broad range of behaviours when providing examples of emotional abuse (e.g. belittling, absence of public affection, criticising military career).

*Sometimes the Mrs will just say comments or try and demean me or belittle my efforts, or try and make me feel bad about myself. […] she told me that everything I have ever achieved since I came to this country is nothing, and she just thinks that I wasted my time in the army […] basically just putting down. [P21; Male Army personnel; IPVA victimisation with retaliation reported]*

Some military personnel struggled to identify examples of abuse despite endorsing its occurrence within their relationship. Others did not identify behaviours as abusive despite descriptions suggestive of emotional or psychological abuse, suggesting a lack of understanding or awareness among military personnel of non-physical forms of abuse.

*What would happen is, if we had an argument, I would get upset. As soon as I got upset, they [my ex-partners] would mock me and put me down, calling me weak and sad and, ‘Oh, you’re crying again,’ that sort of stuff. [P32; Female Army personnel; bidirectional abuse reported]*

*I probably have… just been a bitch. […] It wasn’t all the time. It was probably just getting back at him, if he had said something to me. [P20; Female RAF personnel; Dual military relationship; bidirectional abuse reported]*

All civilian respondents reported experiences of emotional abuse. They described being humiliated, criticised, belittled and put down by their military partners.

He always put me down. He always said, if I ever wanted to leave him that nobody else would ever want me, and I am lucky that he would have me. (P3; Civilian partner of military personnel)

If I said something, it would be, ‘Oh, you’re so stupid. Nobody likes you.’ ‘Why are you wearing that? You look ridiculous.’ ‘Your family don’t even like you.’ ‘There’s no point in leaving me because you would be on your own. (P2; Civilian partner of military personnel)

Perpetration of psychological abuse (e.g. verbal aggression, threats of violence), was reported by some military personnel, including shouting or swearing at their partner. In many cases, this appeared to result from poor emotion regulation and difficulties managing anger or stress. Psychological abuse was a strong theme among military personnel in relationships in which bidirectional abuse occurred. Some personnel reported having experienced unilateral psychological abuse victimisation, with a few reporting retaliation. It was at times difficult to discern the severity of verbal aggression within military personnel narratives, perhaps due to minimization or reluctance to disclose.

*I will be very cruel at times with some of the phrases I will come out with, some of the language that I choose to use. […] When I argue, and I can see myself doing it, I am a very ‘in your face’ type person when I argue. I can’t just sit down and reason something out. […] whenever I get angry about something, I seem to lose all those other core skills and just, like I say, go for the jugular. It is frightening sometimes. [P29; Male Army personnel; IPVA perpetration with victim retaliation reported]*

*I think I lost my rag a couple of times towards the end, when she would shout at me in public. So I would shout back. […] after three hours with someone shouting at you, and you are trying to mediate or try to come up with a solution, it just gets too much sometimes. [P3; Male Army personnel; IPVA victimisation with retaliation reported]*

All civilian respondents reported having experienced psychological abuse. This included verbal abuse and threats to harm, inducing fear. Civilian respondents shared experiences of being blamed for the arguments, aggression or abuse, or thinking they were “*going crazy*”. This was named as “*gas lighting*” by one participant.

He would say, ‘If you do that again, I’ll beat the shit out of you.’ He would say things like, ‘I wish you were a bloke because I could hit you harder.’ He would say, ‘You’re going to get a slap for that later.’ (P8; Civilian partner of military personnel).

He used to threaten me with what he would do to me if I left him as well: ‘I could make it look like suicide,’ and things like this. (P10; Civilian partner of military personnel)

**Controlling behaviours.** Perpetration of controlling behaviours was reported by male and female personnel and was exclusively unidirectional. As with emotional or psychological abuse, military personnel who reported perpetrating controlling behaviours tended to give less explicit or clear-cut examples which varied from reportedly unintentional intimidation to not wanting their partner to dress certain ways or controlling their whereabouts.

*In terms of controlling behaviour, when it comes down to arguing, I think what we sometimes lose sight of is that I am just under six feet which could be intimidating to somebody, and with what I was talking about [in the survey], the controlling, it is not intentionally being controlling; it is more an element of control in the perspective of somebody who is potentially intimidating you. [P15; Male Army personnel; bidirectional abuse reported]*

*If I say to […] my partner, ‘I need you here by 1 o’clock,’ and she is not, I will say, ‘You should have been here by 1,’ and then the next time she will be there by 1. [P8; Male RAF personnel; IPVA perpetration reported]*

Many military personnel reported being victims of controlling behaviour, with male personnel more commonly reporting more severe experiences of controlling behaviours perpetrated by their female partners. In contrast to those reporting perpetration, personnel who reported victimisation provided more definitive examples of coercive control, including limiting visits with others, limiting spending money, controlling decision-making in the household and being pressured to stay home.

*He didn’t like me associating with certain people, even friends if he didn’t think they were the right people. […] He used to try to control what I would wear and things like that as well. [P17; Female RAF personnel; Dual military relationship; IPVA victimisation with retaliation reported]*

The majority of civilian respondents described experiencing controlling behaviours within their relationships with their military partners. This closely mirrored the experiences of military personnel reporting coercive control victimisation, encompassing physical restriction of leaving the home, economic control by limiting their ability to work or manage their finances independently, and emotional control, for example stopping them from seeing family, socialising, or reading communication they have with others.

He would threaten to lock me in the house if I didn’t do what he wanted. He would say to me, ‘I’m going to tie you up and make sure you stay there. You can’t go out’ (P19; Civilian partner of military personnel).

If I wanted money, I had to ask for it. […] If I went to the shops, he had a timer on his watch, and he used to time me from the moment I left the house to the moment I got home, because he thought I was meeting somebody for an affair. (P7; Civilian partner of military personnel*)*

**Physical abuse.** Some military personnel described being exposed to multiple types of abuse (i.e. physical, and emotional/psychological/controlling), although this was more common in the narratives of civilian partners. Among military personnel, physical violence often escalated from verbal aggression. Both male and female personnel reported perpetrating physical violence (e.g. hitting, slapping, pushing), often towards objects but also towards their partner. More severe forms of physical violence perpetration (e.g. punching) were more prominent in reports by male personnel.

*She was getting quite angry at me, and I honestly can’t remember what for, but I was trying to ignore her, and this is going to sound ridiculous to you – I was reading a book, and she grabbed my book and tried to rip it in half, and I just saw red. I lashed out verbally, and she punched me, so I punched her back. [P26; Male Army personnel; bidirectional abuse reported]*

Physical aggression victimisation was less frequently reported by military personnel compared to civilian respondents. Many male and female personnel who reported severe physical victimisation by women described the use of weapons (e.g. gun, knives, poison, cars, vase, glass bottle). Despite the severity of this, male personnel who experienced physical violence victimisation by female partners tended to minimise the severity of the violence they experienced. Female personnel who reported perpetrating physical abuse also showed a greater tendency to minimise their physically violent behaviour towards their partners.

*She was quite volatile. She threw a knife at me one night. That was quite entertaining. [P2; Male RAF personnel; IPVA victimisation reported]*

*Sometimes I would whack him occasionally, but not very often. [P20; Female RAF personnel; Dual military relationship; bidirectional abuse reported]*

In contrast, almost all civilian respondents reported regular, and often severe, physical abuse.

He tried to strangle me, kicked me. He once kicked me out of the house, physically kicked me […] he would pull me by the hair. He would spit in my face and just really throw me around. Once he picked me up by my belt and threw me across the room. (P2; Civilian partner of military personnel)

A minority of female personnel who experienced physical IPVA victimisation shared not feeling able to retaliate due to their partners strength and noted their partner’s denial of perpetration if they were hurt with objects, illustrated below.

*He did throw an iron at me once […] One time I did try and hit him back, and he laughed at me. […] it could just be pushing or shoving me really hard into something. […] It was like, ‘I didn’t hit you. It was that that hurt you.’ […] And in fact it was even my fault for falling in that direction. [P17; Female RAF personnel; Dual military relationship; IPVA victimisation with retaliation reported]*

Similarly, some civilian partners shared that their military partners would deny their role in any harm incurred, especially if this had resulted in them falling or banging against an object, which fed into the psychological abuse.

He threw me up against the wall next to the stairs and went to push me down the stairs, and slammed the door in my face and I got a black eye from it. […] but of course he didn’t touch me; it was the door that hit me, not him. (P18; Civilian partner of military personnel)

He threw me quite violently into our table and chairs, whereby I smashed my face and head on the bench and on the table. […] He said he had never raised a hand to a woman and he didn’t raise a hand to me. In his mind, he hadn’t hit me; the fact that he had caused all this damage. (P14; Civilian partner of military personnel)

**Sexual abuse.** No military personnel reported perpetration of sexual aggression. A small number of male and female personnel reported experiences of sexual coercion and one female personnel reported a serious sexual assault.

*Sometimes she would try and force an issue [sexually] that I didn’t want to do. […] I would just say to her, ‘No, I’m not into that.’ She would try it on, and I would say, ‘No, no, no,’ and then she would push and push. [P26; Male Army personnel; bidirectional abuse reported]*

*He once dragged me across the bedroom floor by my hair […] After he had done that, he then penetrated [me]. [P39; Female Army personnel; IPVA victimisation reported]*

Sexual abuse was more prominent among the narratives of civilian respondents, although again represented a minority. Among civilian respondents who did describe sexual violence, this was increasingly common towards the end of the relationship as violence within the relationship generally escalated. Such behaviours often occurred within the context of wider controlling behaviours and emotional abuse.

As time progressed, he could be quite violent. He raped me more times than I can count. I don’t know. I have lost count of how many times. And would make me perform things and do things that I didn’t want to do. (P23; Civilian partner of military personnel)

If you didn’t sleep with him, he would either sulk, get into an argument so that, when you say no, it is because you have got somebody else. Then, by the time the argument has gone on for two hours, you just say yes anyway just to shut him up. So, I would say it was coercive in making you do something that you don’t want to do […] rather than violently making you do something; wearing you down until you do, […] I would say weekly. (P10; Civilian partner of military personnel)

### Theme 3: Perceived Drivers of Abuse

**Motivations.** Within some military personnel narratives, the motivations behind the abuse they either perpetrated or experienced were perceived to stem from the need to assert power or control. More frequent IPVA was reported in those relationships. In some cases, the apparent need for power or control by both male and female personnel was perceived to be related to military culture and socialisation, such as the need for order and the expectation of subordination, as illustrated in the quote below. Among civilian respondent narratives, the majority described the IPVA they experienced to be more clearly motivated by their military partner’s need to assert power or control.

*[Our relationship nearly broke down mainly] to do with the army and situations. I had to be in control of situations, and the situations had to be under my control. What I said had to happen, and that was it. There was no leeway or anything. [P24; Male Army personnel; IPVA perpetration with victim retaliation reported]*

I wasn’t allowed to be who I wanted to be. I had to be moulded into what he wanted. […] I wasn’t allowed to wear what I wanted to wear. I wasn’t allowed to cut my hair. I wasn’t allowed to go anywhere without asking him. […] I wasn’t allowed to learn to drive and then I wasn’t allowed my own car. Sometimes he would hide the door keys from me so I couldn’t go out of the house. (P19; Civilian partner of military personnel*)*

For other military personnel, motivations for the abusive behaviours stemmed from the expression of negative emotions, such as anger; the expression of broader dissatisfaction within the relationship; and poor communication skills. For a minority of personnel, motivations for IPVA were less clear.

*I would get really angry and mad. What triggered me to go for therapy is when I punched her. [P21; Male Army personnel; IPVA victimisation with retaliation reported]*

**Triggers.** Military personnel and civilian respondents reported both internal and external triggers for escalations in conflict and IPVA behaviours in their relationships. Internal triggers for perpetration of abuse reported by both military personnel reporting perpetration or participants reporting victimisation included jealousy, mistrust, mental health difficulties, low self-esteem and insecurities.

*She didn’t like me going to see my own family. […] Before I was going to see a friend or a family member, and she wasn’t coming with me, she would stage some massive […] break down or an argument or something, […] it was basically like, ‘If you leave me now, that’s it,’ […] she always used to do that so that I wouldn’t be able to go. […] I think it was just an insecurity thing. [P17; Female RAF personnel; Dual military relationship; IPVA victimisation with retaliation reported]*

External triggers for perpetration of abuse reported by participants included stressors or disagreements relating to work (primarily military careers but also post-Service employment), parenthood, military-related separations or high expectations of personnel for structure and order in the home.

*The slightest thing [could make me lose my temper] – it could be that I get home and say, ‘The bins haven’t been put out,’ and that was it; I would fly off the handle. So simple, minor, petty little things that don’t really matter. […] it would happen and I would just see red and just go off shouting. [P24; Male Army personnel; IPVA perpetration with victim retaliation reported]*

Some participants described perpetration of abuse by military personnel to be heightened upon returning from deployment. Civilian respondents also observed an increase in abuse when their military partner transitioned out of Service.

The actual physical violence, when he started actually turning onto me [rather than walls/objects], was when he returned from his tour. […] He had a really short fuse, he had no tolerance for anything. He just seemed to just get angry really fast, and, again, it didn’t seem to be a problem to take it out on me now. (P3; Civilian partner of military personnel)

I would say he [his aggression] got worse when he left the army. (P10; Civilian partner of military personnel)

Military personnel and civilian respondents both reported that abuse escalated in the context of alcohol use, although this was more prominent in the narratives of civilian respondents.

He tried to strangle me, kicked me. He once kicked me out of the house, physically kicked me, and I landed on next door’s doorstep opposite in a ball. He would pull me by the hair. He would spit in my face and just really throw me around. Once he picked me up by my belt and threw me across the room. […] [Triggers were] usually alcohol. Actually every time it was alcohol. (P2; Civilian partner of military personnel)

Civilian partners additionally identified changes of circumstance, such as being pregnant or moving in together, as triggers for their experiences of abuse worsening.

I never really saw that side of him until we lived together. So it didn’t happen frequently until, we moved in together when I was pregnant and I would say that it got ten times worse quite quickly; as soon as we moved in together. (P21; Civilian partner of military personnel)

Some participants who experienced victimisation identified that social isolation, dependency on their partner and/or their mental health difficulties played a role in increasing their vulnerability to abuse. Military related relocations were described by civilian respondents to increase their isolation from their support networks and their financial and emotional dependency on their military partner.

When we moved [overseas], [our relationship] all changed. I obviously gave up my tenancy, I gave up my job, I gave up my friends. So I was solely dependent on him. […] For me to suddenly be needy of him, I think he quite liked that. And a lot of his friends were like that […]‘My wife’s not allowed to do that,’ or, ‘I wouldn’t let my wife do that’. (P23; Civilian partner of military personnel)

Mental health difficulties were a more prominent factor in the narratives of military personnel who experienced IPVA victimisation than civilian respondents, especially among male personnel who deployed, as illustrated in the quote below.

*[After deployment], that is when I started getting nightmares and started withdrawing, just not talking about feelings and all that sort of thing. I guess that just allowed, with me being not very verbal and outspoken about what I was feeling, her being extremely outspoken about how she was feeling, it just allowed her to be controlling in that respect and get what she wanted because I was never bothered enough about it. [P13; Male Army personnel; victimisation with retaliation reported]*

#### Conflict resolution

Most military personnel described poor conflict resolution skills, leading to the perpetuation of tensions and at times, escalations in violence. This was especially prominent in the narratives of male and female personnel in relationships in which bidirectional abuse occurred. Most often, military personnel described responding to conflict by walking away or with matched aggression. Most civilian respondents described similar experiences of ongoing and unresolved conflict with poor conflict resolution, attributing blame to their military partner, and reporting minimisation or escalations in violence if they confronted their military partner’s abusive behaviours.

*Sometimes, when she argues back and holds her ground, that can quite often antagonise me more. Usually on those occasions, I think when I then stand my ground and it is almost like two bulls crashing together, with me being more aggressive. [P29; Male Army personnel; IPVA perpetration reported]*

You wouldn’t resolve [conflicts]. He would do something, I wouldn’t dare say anything, and then he would pretend like nothing had happened. I knew that, if I did try and bring it up or ask for an explanation, it would just start it all again. (P20; Civilian partner of military personnel)

### Theme 4: Perceived Impact of Abuse

**Physical health.** Some military personnel, especially female, reported physical injuries as a result of the IPVA they experienced. These tended to be superficial injuries such as bruising and scratches, with no long-term impacts reported. In contrast, many civilian respondents reported physical trauma as a result of the IPVA they experienced by their military partners, ranging from sprains to more severe and enduring injuries, such as fractures, broken bones or disfigurement.

*I used to always have bruises of her fingerprints. She would just be holding my arms, like holding me away from wherever I was trying to go, holding me up against the wall, even down on the bed so that I didn’t move and couldn’t leave. [P17; Female RAF personnel; Dual military relationship; IPVA victimisation with retaliation reported]*

He has strangled me until I have actually collapsed on the floor and been unconscious. He has bashed my head and it has made a hole in the wall. He kicked me hard I am pretty sure I broke my collarbone, but I never went to the doctor’s. […] I couldn’t leave the house afterwards. I would be locked in until I was better, so no one could see me and realise that we weren’t the perfect couple. (P12; Civilian partner of military personnel)

**Mental health.** A minority of military personnel reported IPVA experiences to negatively affect their or their partner’s psychological wellbeing, at times amplifying pre-existing difficulties.

*He just used to come out with stuff and just make me feel small and stupid. I look back now, it sounds daft, and excuse me for crying, but I have done a lot of thinking since we split up. […] I think the doctor said I was depressed. [P20; Female RAF personnel; Dual military relationship; bidirectional abuse reported]*

*Like I say, the way she described it in her own words was, ‘I’m a shell of the person I used to be. You’ve broken me right down.’ That was the point where she went to the doctor’s and was put on antidepressants. I actually pushed her into depression. [P24; Male Army personnel; IPVA perpetration with victim retaliation reported]*

Military personnel who reported more severe experiences of IPVA victimisation described a greater impact on their mental health. The latter mirrored narratives of civilian respondents, who described acute and chronic mental health difficulties stemming from the abuse they experienced and their continual state of fear and submission. These included but were not limited to, mental difficulties and disorders such as Post Traumatic Stress Disorder (PTSD), anxiety, low mood, difficulties with trust and poor self-esteem.

As the abuse escalated and spiralled, I became more scared of him and tried to just not lose my temper and just hide from him or stop him from blowing up to start off with. (P19; Civilian partner of military personnel)

I had extreme anxiety, I had treatment for CPTSD – Complex PTSD myself, because of all the years of abuse. […] I was numb. I didn’t get angry, I didn’t get sad, I didn’t really get happy. I just dissociated myself, and I was just like this empty shell of a thing. (P12; Civilian partner of military personnel)

**Impact on parenting and children.** While **s**ome military personnel noted that their children would witness their relationship conflict, only a minority shared that their children were affected by the IPVA in their relationships. For example, some children were described to have developed difficulties relating to mental health or anger management. Some military personnel recognised that their controlling behaviour and high expectations also extended to their children.

*My eldest daughter witnessed when [my partner] tried to stab me with a carving knife. So that has been difficult for her. [P40; Male RAF personnel; IPVA victimisation reported]*

*My husband will still now often say to me, ‘You’re not in the bloody army now.’ He says that I treat my children like a general would treat their soldiers. [P33; Female Army personnel; bidirectional abuse reported]*

In contrast, all civilian respondents with children noted how their children were witnesses to violence within the household, with a few noting their military partners were aggressive towards their children. The reported consequences for children of civilian respondents were primarily the development of psychological difficulties, such as low mood and anxiety or PTSD symptoms, but also included increased aggression and school-related difficulties.

What he did do was then he would hit the children or grab the children by the throat or hair. He was physically abusive to the children. (P23; Civilian partner of military personnel)

All my children, not so much the youngest one, but my daughter and my eldest son, even now they have counsellors and are under CAMHS [Child and Adolescent Mental Health Services]. (P11; Civilian partner of military personnel)

Civilian respondents also perceived the psychological and physical injuries sustained as a result of IPVA perpetrated by their military partners to impair their ability to parent and protect their children. Civilian respondents reported impaired functioning across a number of parenting domains, such as increased levels of parenting stress, which impacted on the quality of parent-child relationships. Emotional neglect, overt hostility and controlling behaviours towards their children by their partners, mirroring the IPVA within their intimate relationship, were also reported.

My mental health had a huge impact on my children because their mother was constantly depressed. […] I look back to their childhood and I have lots of regrets where they were neglected, they weren’t played with, they were shouted at, they were physically abused. (P23; Civilian partner of military personnel)

**Occupational and social functioning.** A minority of military personnel reported impacts on their occupational or social functioning, including direct impact on their work and feeling isolated, as illustrated below. A few female personnel described their experiences of IPVA victimisation to impact on their social functioning secondary to the impact on their mental health and self-esteem, affecting how they relate to and perceive others.

*We had a big argument. I didn’t go into work the next day because it really affected me. […] I felt as though then my mental health was affected because I felt a bit isolated; I didn’t know who to speak to. [P1; Male Navy personnel; bidirectional abuse reported]*

*I had had my confidence really knocked, I just stopped being the person that I was. […] [It is] quite difficult for me to trust somebody initially. I tend to try not to be too connected to somebody too quickly, but I also find that, if somebody said that they loved me, […] I find it difficult to accept that that might be genuine. [P39; Female Army personnel; IPVA victimisation reported]*

Impacts on occupational and social functioning were more apparent among civilian respondents, who described difficulties finding or maintaining employment or cultivating friendships as a result of the coercive control perpetrated by their military partners or needing to conceal physical injuries.

I had been isolated from pretty much all my friends and family, and I think, because of my fear of him finding out, nobody really knew. (P15; Civilian partner of military personnel)

I had to have time off work due to bruising and bits like that or there would be days where he wouldn’t want me to go in. Ultimately, each time I had to give up my job because it was getting my point where I would be going to get sacked because I wasn’t going in or I gave the job up. (P19; Civilian partner of military personnel)

## Discussion

This research used qualitative data to explore the IPVA experiences of serving and ex-serving UK military personnel as well as civilian partners of serving or ex-serving military personnel: data on IPVA experiences of UK serving and ex-serving military personnel, recruited from a sample who had previously endorsed IPVA perpetration or victimisation in a self-administered survey (MacManus et al., 2022; Stevelink et al., 2018), and data from civilian victim-survivors of an abusive relationship with a military person. Four main themes were identified, describing the patterns of IPVA perpetrated or experienced by participants, the types perpetrated or experienced, the perceived drivers of IPVA and the perceived impact of IPVA.

Most serving and ex-serving military personnel in the sample reported bidirectional abuse or unilateral IPVA victimisation within a relationship. The high levels of bidirectional abuse resonates with findings from quantitative data in both civilian and military samples (Langhinrichsen-Rohling et al., 2012b; MacManus et al., 2022; Park et al., 2021). This highlights the importance of recognising the potential role of both partners in violent relationships, though the greater impact and likelihood of injury among females resulting from male violence perpetration, and the potentially different mechanisms for men and women in pathways to victimisation and perpetration is acknowledged (Archer, 2002). Bidirectional abuse was especially prominent in those reporting dual military relationships, a group currently under-represented in the military literature. The impact of such ‘toxic relationships’, in which bidirectional abuse occurs, on the individual partners as well as others in the household, especially children, is not sufficiently recognised (Domoney & Trevillion, 2021; Zemp et al., 2016). A minority of serving or ex-serving military personnel reported unilateral perpetration of abuse. Minimal gender differences in IPVA experiences among serving and ex-serving military personnel were observed in patterns and types of IPVA, though reports of severe IPVA perpetration were more prominent among male serving or ex-serving personnel. This broadly supports our quantitative findings, that male serving or ex-serving personnel were significantly more likely to report perpetration of emotional, psychological and controlling abusive behaviours than their females counterparts (MacManus et al., 2022). Civilian respondents were recruited as victim-survivors of abuse perpetrated by a military (ex)partner and all described experiences of moderate to severe unilateral victimisation, mostly motivated by the need for their serving or ex-serving military partner to assert power and control, and some which would have been appropriately labelled Intimate Terrorism (Johnson et al., 2014). Although these findings do not capture IPVA perpetration by civilian partners of military personnel (Park et al., 2021), they resonate with civilian partner experiences of IPVA perpetrated by military personnel found in other studies (Gray, 2016; Williamson, 2012) and supplement the military personnel narratives which appeared prone to under-disclosure of perpetration.

Various motivations for perpetrating IPVA have been identified in the literature, including power/control, communication difficulties and jealousy (Johnson, 2006; Langhinrichsen-Rohling et al., 2012a). Although a minority of serving or ex-serving military personnel described experiences of perpetration or victimisation motivated by power or control, for many the abuse followed patterns of situational couples violence seemingly fuelled by negative intrapersonal factors (e.g. anger, mental health difficulties), interpersonal factors (e.g. relationship dissatisfaction) and difficulties with communication (Johnson, 2006; Langhinrichsen-Rohling et al., 2012a). These motivations align with the prevalent reporting of psychological abuse, especially verbal aggression, among military personnel. The findings demonstrate the significance of emotion dysregulation and poor communication skills in IPVA experiences, showing some support for the frustration-aggression hypothesis, whereby increased emotional arousal, particularly anger, may explain the association between work-family conflict or marital discord and IPVA (Park et al., 2021). In addition, poor conflict resolution was described in both serving and ex-serving military personnel and civilian respondent narratives, at times perpetuating the abuse, highlighting distinct areas for interventions to target. Poor conflict resolution was especially prominent in relationships in which bidirectional abuse took place, suggestive of mutually dysphoric or retaliatory violence (Langhinrichsen-Rohling, 2010).

In line with research among civilian samples (Bell & Orcutt, 2009; Oram et al., 2013), individual level risk factors such as poor emotion regulation, mental health difficulties and alcohol use were described by participants as significant drivers of IPVA perpetration. Some participants described how military socialisation and the culture of high expectations of order and a need for control permeated their homes and were perceived to provide significant context for abuse perpetration, as frequently outlined in the literature (Bradley, 2007; Trevillion et al., 2015). This supports theories of top-down cultural and institutional influences on violence and aggression, as outlined in the Ecological Framework of violence (WHO, 2002), in which the role of society or societal institutions in impacting systems, attitudes, and beliefs at community, interpersonal and individual levels to influence whether violence is encouraged or inhibited is acknowledged. The prevailing military cultural norms described by participants, such as those around male dominance and hierarchy and the endorsement of violence as an acceptable method to resolve conflicts, are potent examples of how effective partner violence prevention strategies must consider more than individual risk factors.

Serving and ex-serving military personnel, and especially civilian respondents, identified high risk periods for IPVA, including the peri-deployment period and transitions out of Service. Post-deployment mental health was identified as a trigger for perpetration by both perpetrators and victim-survivors within the sample, corroborating research which has found deployment-related trauma to be associated with IPVA perpetration (Kwan et al., 2018, 2020; Lane et al., 2022; MacManus et al., 2022). Poor mental health in the period following deployment was an especially prominent trigger for IPVA victimisation identified by male personnel, supporting previous quantitative research findings (Sparrow et al., 2017, 2020). The complex role of mental health difficulties in creating a context in which both perpetration and victimisation of abuse is more likely to occur warrants further exploration. Our findings suggest that internalising behaviours related to mental health difficulties (e.g. withdrawal) may increase vulnerability to abuse, whilst externalising behaviours (e.g. losing temper) may be more closely related to, and perhaps drive, IPVA perpetration. Civilian respondents identified isolation from peers following military related relocations as increasing their dependency on their military partner and, hence, vulnerability to abuse. This echoes previous research (e.g. Kern 2017) and is notable in understanding how military factors influence experiences of abuse among non- or ex-military partners and subsequent help-seeking. It is possible that factors relating to poor mental health and isolation are of particular significance in the context of the COVID-19 pandemic due to local lockdown rules and social distancing, travel limitations and other stressors such as financial instability.

Participants described a range of negative consequences of IPVA within their relationships, corroborating existing research findings in both civilian and the Armed Forces communities. These included effects on their physical and mental health (Campbell, 2002), their ability to parent and children’s wellbeing (Christie et al., 2019; Jouriles & McDonald, 2015), and their ability to gain and maintain employment and relationships (Hines & Douglas, 2018; Johnson et al., 2014). Negative impact was reportedly greater and long-lasting within civilian respondent accounts. While the abuse experienced by serving or ex-serving military personnel compared to civilian respondents may have overall been reported as less severe and to have had a less significant impact, there may also have been a tendency for military personnel to minimise relationship difficulties and abuse. Less reporting of perpetration and the lack of clear examples of abusive behaviours perpetrated by military respondents, as well as the discrepancies in military respondent reporting identified between the KCMHR survey and within the interviews, may be driven by tendency for non-disclosure of perpetration and social desirability bias, as observed in civilian literature (Caetano et al., 2002). This may also be the case for those reporting victimisation, illustrated by the tendency for military personnel to minimise physical IPVA perpetrated by female partners. Indeed, military culture has been argued to encourage stoicism through machismo and promote legitimisation of violence within military contexts (Bradley, 2007; Trevillion et al., 2015), which could spill over into family spheres and may impact on insight and disclosure. These factors may also have impacted personnel awareness and insight of IPVA, and affected the reporting of drivers for abuse and impact, which were clearer and more prominent in the narratives of civilian respondents.

Methodological differences, especially in recruitment, may account for the differences between the accounts of serving and ex-serving military personnel and civilian respondents. As civilian partners were largely recruited from support services, it is likely they have experienced more severe forms of IPVA given the greater propensity to seek help from services for IPVA (Coker et al., 2000; Goodman et al., 2003) and may be better able to reflect on and articulate their experiences. It remains likely that serving and ex-serving military personnel who engage in or have experienced more severe IPVA motivated by power or control may be less inclined to participate than personnel with less severe experiences. Nevertheless, the purpose of this research was not to compare but to use the samples to complement one another and provide a fuller understanding of experiences of IPVA within couples in which one or both partners are serving or ex-serving military personnel. Unfortunately, subgroup analysis (for example by rank, serving status, and engagement status) was limited across datasets due the complexity of the IPVA and military characteristics data, for example many military personnel respondents reported different relationships with differing IPVA patterns. Further qualitative investigation is warranted to explore differences in experiences across groups. In addition, the present research recruited a predominantly White British sample and only a limited number of female serving or ex-serving military personnel. We were also limited in our observations of gender and sexual orientation given only two military personnel respondents reported same-sex relationships and civilian respondents were all female in heterosexual relationships. More research is required to explore experiences of dual military couples, LGBT couples, civilian partners of other genders and those from ethnic minority backgrounds in more depth.

There is currently a tendency for IPVA interventions to consider perpetrators and victim-survivors separately (Bates, 2016). While we recognise the appropriateness of such an approach in some cases, our findings of frequent bidirectional abuse within military and veteran relationships demonstrate that the reliance on a rigid victim/perpetrator binary is limited for some, stressing the need for programmes to acknowledge the bidirectionality of abuse in some relationships and also the need to work with couples. We acknowledge that this can present issues for risk management (e.g. Kohn 2010; Simpson et al., 2008), but a growing body of evidence now suggests that working with both partners can be very effective (Taft et al., 2016), e.g. when motivations for help occur within the context of parenting (Domoney & Trevillion, 2021). Furthermore, our findings highlight a need for perpetrator programmes to be tailored to female as well as male partners. Although some victim-survivor support services do cater for men, our findings highlight the need for more support services for male victim-survivors and services that have a specialist understanding of the military context. The present findings suggest a lack of understanding of how abuse may present, particularly non-physical forms of IPVA, stressing a need for training and awareness among personnel to support the identification of abuse within relationships where one or both partners are serving or have serviced in the Armed Forces. In addition to being a trigger for perpetration, personnel mental health and alcohol use was perceived to be an important trigger for IPVA victimisation within our sample, especially among male personnel who deployed, and the rich narratives shed light on the nuanced mechanisms underlying these risk factors. Considering the often unidimensional findings from quantitative research in this area, which have highlighted associations between mental health problems and both perpetration and victimisation (e.g. Kwan et al., 2020; MacManus et al., 2022; Sparrow et al., 2020), the current results highlight the complex relationship between IPVA and mental health problems which requires better understanding and more nuanced public discourse. Improved awareness and identification of mental health problems within serving and ex-serving military personnel samples may help mitigate against abuse.

This research explored IPVA experiences from the perspectives of serving and ex-serving military personnel who reported IPVA perpetration or victimisation and civilian victim-survivors of abusive relationships with serving or ex-serving military personnel. Although these findings highlight the severe nature of abuse experienced by civilian partners of serving or ex-serving military personnel, characteristic of intimate terrorism, many military personnel respondents described bidirectional IPVA, often arising from situational couples violence, and which was often normalised or not identified as abuse. The results suggest that military culture and context play an important role in the occurrence of IPVA in couples in which one or both partners are serving or ex-serving military personnel, which warrants further exploration. Our findings also highlight the need to consider limitations of the often gendered victim/perpetrator binaries common in interventions and services, and the role of emotion dysregulation, poor communication skills and mental health difficulties in explaining and perpetuating abuse.
